# Screening and Selection of Hypoallergenic Buckwheat Species

**DOI:** 10.1100/tsw.2002.157

**Published:** 2002-03-27

**Authors:** Arun Nair, Taiji Adachi

**Affiliations:** Kiyomoto Bio Co. Ltd., 2-28-4 Sakura, Gakuen Kibanadai, Miyazaki 889-2154, Japan; Lab of Plant Genes and Physiology, College of Agriculture, Osaka Prefecture University, 1-1, Gakuen-Cho, Sakai, Osaka 599-8531, Japan

**Keywords:** allergenic proteins, buckwheat, PCR, SDS-PAGE

## Abstract

Both common buckwheat (*Fagopyrum esculentum*) flour and meal cause an allergy in sensitive patients, and if unnoticed, it can be fatal. It has become a potential occupational hazard for some mill workers. The development of hypoallergenic buckwheat would be more efficient if natural mutants for allergenic protein are detected. A screening and selection method was developed using SDS-PAGE coupled with PCR techniques. SDS-PAGE analysis of 14 different species of buckwheat revealed that *F. lineare* and *F. urophyllum* lack the 22-kDa major allergenic protein. PCR-based screening with specific primers for sequences encoding the allergenic protein was also effective in distinguishing the allergen-deficient species.

## INTRODUCTION

Sodium dodecyl sulphate polyacrylamide gel electrophoresis (SDS-PAGE) of seed proteins has been extensively used to distinguish cultivars and species of different plants. The basic principle of evaluation is qualitative banding differences, i.e., the presence/absence of a band of a particular molecular mass. SDS-PAGE of bulked seed protein samples has proven to be an effective method for distinguishing cultivars of largely outbreeding grasses, legumes, and sugar beets[1,2,3,4,5]. In buckwheat, too, several reports are available. Intravarietal heterogeneity for different protein subunits was reported[6], and cultivar identification based on total seed proteins was proposed[7]. The same principle has been extended to screening different buckwheat species for the presence or absence of the major allergenic protein. A 22-kDa protein was earlier reported as the major allergenic protein in buckwheat[8].

In another approach, a simplified method based on polymerase chain reaction (PCR) was developed to screen buckwheat species for the gene encoding the allergenic protein. PCR has been used in various studies for the rapid, sensitive, and reliable detection of many pathogens: badna viruses[9], banana streak viruses[10], and *Yersinia enterocolitica* from various foods that cause yersiniosis[11].

In our experiment, after SDS-PAGE of buckwheat seed proteins, *Fagopyrum* species, which showed an absence of the 22-kDa band, or where the band was difficult to differentiate, was selected for PCR amplification. The amplified products were sequenced to confirm the specificity. The objective of this study was to develop fast and reliable screening methods to select buckwheat lines without allergenic proteins.

## MATERIALS AND METHODS

### Protein Extraction and Electrophoresis

Common buckwheat (*F. esculentum*) cultivar “Miyazaki zairai” was field grown at Miyazaki, and seeds were harvested before being subjected to electrophoresis. (The seeds of the remaining 13 *Fagopyrum* species were kindly provided by Dr. Campbell, Kade Research Ltd., Manitoba, Canada.) Before sampling each species, 50 seeds were bulked and homogenized. For some rare species whose seed availability was limited, only 10 seeds were homogenized as the bulk sample. Protein extraction and electrophoresis were carried out as per Nair and Adachi[8].

### DNA Extraction

Genomic DNA was extracted from 0.5–2.0 g of fresh young leaves of *F. esculentum*, *F. gracilipes,* and *F. lineare*. The leaf homogenates were washed 3–5 times with washing buffer (0.1 M HEPES, 0.1% (w/v) polyvinyl pyrollidone, 4% (v/v) 2-mercaptoethanol, pH 8.0) before isolation. The DNA was isolated in DNA isolation buffer (0.3 M NaCl, 0.5 M Tris-HCl, pH 7.5, 20 mM EDTA, pH 8.0, 1% (w/v) SDS, 10 mM 2-mercaptoethanol, and 5 M urea). After incubation for an hour, DNA was extracted from the equivalent of phenol-chloroform (1:1). The extracted DNA was washed in ethanol, air dried, and dissolved in sterile distilled water or TE buffer.

### Polymerase Chain Reaction

For PCR analyses, three primers were synthesized to amplify the gene encoding the major allergenic protein (BWA 17 – 5’-CCT TCT CAC TGT GCC TTA TGG, BWA 18 – 5’-GGA TTG GAG CAA GCG TTC TGC, and BWA 20 – 5’- TCC TTC TCA TCT CGG GAC TGG, Life Technologies). PCR amplification was carried out in a final reaction volume of 25 μl containing 1 μl of DNA solution, 10 pmol each of the primers, 0.2 mM dNTPs, 1x PCR buffer, and one unit *Taq* DNA polymerase (Takara). Amplification parameters were 94°C for 5 min, followed by 35 cycles with 94°C for 1 min, 59°C for 2 min, and 72°C for 3 min. A 72°C incubation for 10 min as a final extension step was included. The annealing and extension step in the PCR cycle was given maximum time to increase the fidelity. Amplified DNA fragments were electrophoresed on 1% agarose ethidium bromide gel and observed under ultraviolet light.

### Cloning and Sequencing of PCR Products

The PCR product was directly ligated into a pGEM-T Easy vector (Promega) and transformed using JM 109 high-efficiency competent cells. One microliter of the fresh PCR product was ligated into pGEM-T Easy vector and transformed as per the manufacturer’s instructions. Cells were plated and grown overnight on ampicillin, blue/white selection dishes at 37°C. White colonies were selected, and the plasmid DNA was isolated using a QIAprep Miniprep kit (Qiagen). Positive clones carrying a plasmid with an insert size of approximately 700 base pairs

(bp) were selected for sequencing procedures. DNA sequence analysis of the selected plasmids was performed on an ABI prism 310 Genetic Analyser (PE Applied Biosystems). Sequence reaction was carried out using Big Dye Terminator RR mix diluted with *half*BD (Genpak Ltd.). The reaction product was ethanol precipitated and dissolved in Template Suppression reagent, boiled for 2 min, and cooled on ice before sequencing.

## RESULTS

Total seed proteins of 14 different *Fagopyrum* species were analyzed by SDS-PAGE. The bulk seed samples showed a specific banding pattern with respect to each species. Although the samples were not quantified using specific assay, 2 mg of bulked sample was used for the extraction of proteins. The relative intensity of the band reflects the concentration of a particular protein in each sample. As regards the 22-kDa major allergenic protein, only two species, *F. lineare* and *F. urophyllum*, showed the absence of the major allergenic protein (Fig. 1 and Fig. 2).

Three specific primers were derived from the known buckwheat cDNA sequence encoding the 22-kDa protein. The first set, BWA 17 and BWA 20, were used to amplify more or less the entire length of the cDNA encoding the major allergenic protein: Fag e 1. The second set of primers, BWA 18 and BWA 20, were designed to amplify the gene encoding the 22-kDa protein. cDNA encoding 22-kDa protein of *F. esculentum* and genomic DNA of different *Fagopyrum* species were amplified with the same primer sets. The expected size of the amplified cDNA fragment with BWA 17 and BWA 20 was 1.57 kb and with primer sets BWA 18 and BWA 20 was 0.55 kb. As shown in Fig. 3, the primer set BWA 17 and BWA 20 amplified the cDNA that appeared as a single 1.57-kb band, and the primer set BWA 18 and BWA 20 amplified the cDNA that appeared as a single 0.55-kb band. These results confirmed that the primer pairs were suitable for amplification of the target sequence.

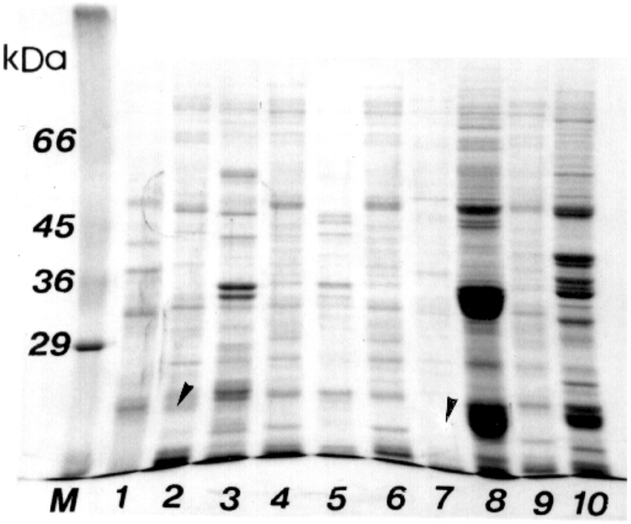

**FIGURE 1.** SDS-PAGE electrophoretogram of seed proteins of different species of buckwheat. Lane 1: *F. esculentum*; Lane 2: *F. gracilipes*; Lane 3: *F. callianthum*; Lane 4: *F. statice*; Lane 5: *F. rubufobium*; Lane 6: *F. macrocarpum*; Lane 7: *F. lineare*; Lane 8: *F. cymosum*; Lane 9: *F. pleioramasum;* and Lane 10: *F. esculentum ancestralis*. The arrowheads indicate the position of the 22-kDa protein band.

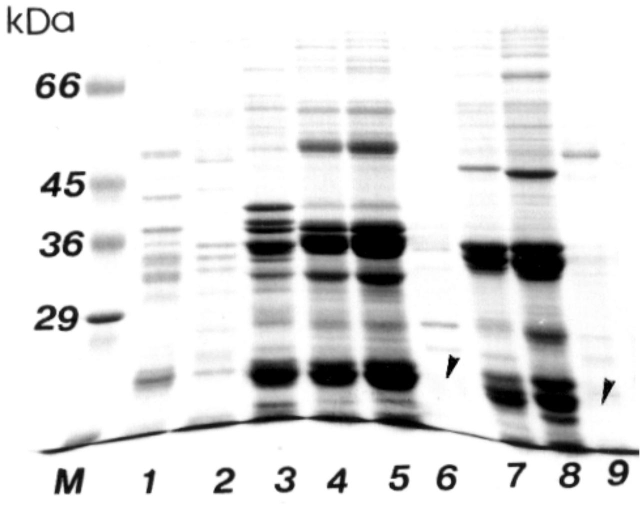

**FIGURE 2.** SDS-PAGE electrophoretogram of seed proteins of different lines and species of buckwheat. Lane 1: *F. esculentum*; Lane 2: *F. capillatum*; Lane 3: *F. homotropicum* #1; Lane 4: *F. homotropicum* #2; Lane 5: *F. homotropicum* #4; Lane 6: *F. lineare*; Lane 7: *F. tataricum* – potany; Lane 8: *F. tataricum*-cm 198; and Lane 9: *F. urophyllum*. The arrowheads indicate the absence of the 22-kDa protein.

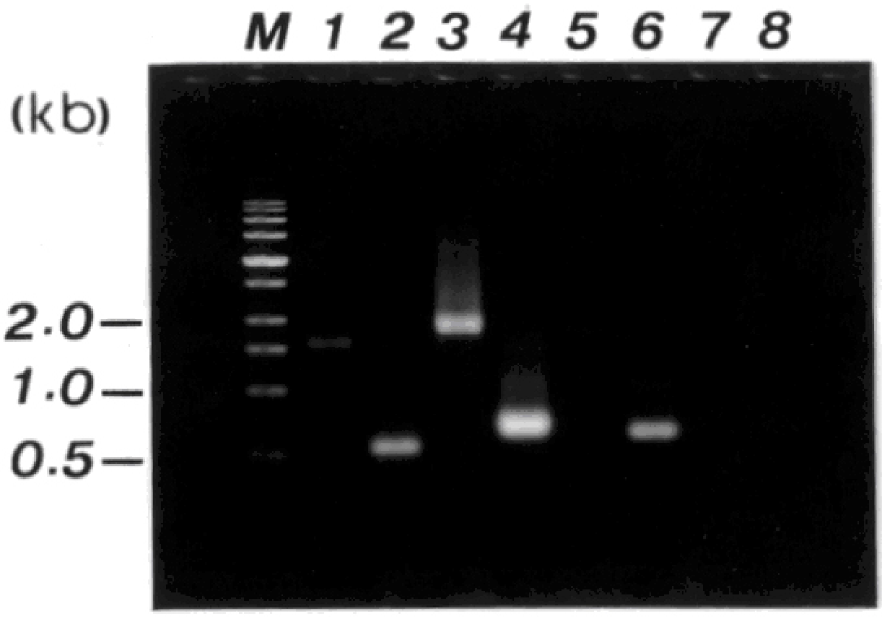

**FIGURE 3.** PCR analysis and screening of buckwheat species for the major allergenic protein. Two specific primers were used to amplify the full length as well as the region encoding the 22-kDa major allergenic protein of the gene *FAGAG1*. Fragments of 1.57 and 0.55 kb were obtained after amplifying the cDNA. The corresponding fragments were 1.8 and 0.7 kb, with genomic DNA of buckwheat, suggesting the presence of introns. M: Molecular size marker, Lanes 1 and 2: cDNA of *Fagopyrum esculentum*; Lanes 3 and 4: genomic DNA of *F. esculentum*; Lanes 5 and 6: genomic DNA of *F. gracilipes*; Lanes 7 and 8: genomic DNA of *F. lineare*.

In case of genomic DNA of *F. esculentum* and *F. gracilipes*, the primer set BWA 17 and BWA 20 amplified and produced one single band of 1.8 and 0.7 kb, respectively (Fig. 3). The slight increase in the size of amplified product indicates that the gene encoding the major allergenic protein has intron sequence(s). In the case of *F. lineare*, the two primer sets did not amplify the genomic DNA (Fig. 3).

```
         10        20        30        40        50        60
GGATTGGAGCAAGCGTTCTGCAACCTGAAATTCAGGCAAAATGTTAACAGGCCTTCTCGC
G  L  E  Q  A  F  C  N  L  K  F  R  Q  N  V  N  R  P  S  R
         70        80        90       100       110       120
GCCGACGTCTTCAACCCACGCGCCGGTCGTATCAACACCGTAGACAGCAACAATCTCCCG
A  D  V  F  N  P  R  A  G  R  I  N  T  V  D  S  N  N  L  P
        130       140       150       160               180
ATCCTCGAATTCATCCAACTTAGCGCCCAGCACGTCGTCCTCTACAAGGTAATGTAAACC
I  L  E  F  I  Q  L  S  A  Q  H  V  V  L  Y  K
        190       200       210       220       230       240
CCAGCTGAAGCCCTAAAAGCGATTGAACTTCGTATTGAAATTCAAGACCTAAAATCGACA
        250       260       270       280       290       300
AATCTGCAAAGTGAAGCTTGATTAACTTCAGATTGACGATAATGCGTTGAATTTATGCAG

310       320       330       340       350       360
AATGCGATCCTCGGACCGAGATGGAACTTGAACGCGCACAGCGCACTGTACGTGACAAGA
N  A  I  L  G  P  R  W  N  L  N  A  H  S  A  L  Y  V  T  R
        370       380       390       400       410       420
GGAGAAGGAAGAGTCCAAGTTGTGGGAGATGAAGGAAGAAGTGTGTTCGACGACAACGTG
G  E  G  R  V  Q  V  V  G  D  E  G  R  S  V  F  D  D  N  V
        430       440       450       460       470       480
CAGCGAGGACAGATCCTTGTGGTCCCACAGGGATTCGCAGTGGTGTTGAAGGCAGGAAGA
Q  R  G  Q  I  L  V  V  P  Q  G  F  A  V  V  L  K  A  G  R
        490       500       510       520       530       540
GAAGGACTGGAGTGGGTGGAGTTGAAGAACGACGACAACGCCATAACCAGTCCGATTGCC
E  G  L  E  W  V  E  L  K  N  D  D  N  A  I  T  S  P  I  A
        550       560       570       580       590       600
GGGAAGACTTCGGTGTTGAGGGCGATCCCTGTGGAGGTTCTTGCCAACTCCTACGATATC
G  K  T  S  V  L  R  A  I  P  V  E  V  L  A  N  S  Y  D  I
        610       620       630       640       650       660
TCGACGAAGGAAGCTTTCAGATTGAAGAATGGGAGGCAGGAGGTTGAGGTCTTCCGACCA
S  T  K  E  A  F  R  L  K  N  G  R  Q  E  V  E  V  F  R  P
        670       680       690       700       710
TTCCAGTCCCGAGATGAGAAGGAGAGGGAGCGTTTCTCCATAGTTTAA
F  Q  S  R  D  E  K  E  R  E  R  F  S  I  V  *
```

**FIGURE 4.** Nucleotide and deduced amino acid sequence of the genomic DNA of *F. esculentum* encoding the major allergenic protein: Fag e1. The forward and reverse primers used to amplify the genomic DNA are underlined. The intron sequences (130 bp) are in bold letters. The arrows indicate the characteristic GU…AG dinucleotide combinations at the intron boundaries.

In order to confirm that the amplified products are actually the targeted products, the PCR products of 0.7 kb from both *F. esculentum* and *F. gracilipes* were directly ligated and transformed. The plasmids were isolated, and DNA sequence analyses were carried out.

The *F. esculentum* genomic DNA encoding the major allergenic protein showed exactly the same nucleotide sequence as that of cDNA except for a 130-bp intron sequence (Fig. 4). In the case of *F. gracilipes* genomic DNA, although the sequence showed a similarity to *F. esculentum*, there were differences in their internal nucleotide sequences (Fig. 5). This was further clarified through multialignment analysis (Fig. 6). *F. gracilipes* also showed an intron sequence of 80 bp, which was shorter than that of *F. esculentum*. The DNA sequence analyses also confirmed that the PCR primers selected to amplify the genomic DNA could be practically used to screen buckwheat plants for allergenicity.

```
        10        20        30        40        50        60
GGATTGGAGCAAGCGTTCTGCAACCTTAAATTCAGGCAAAACGTGAACAACCTGCTCGCG
G  L  E  Q  A  F  C  N  L  K  F  R  Q  N  V  N  N  L  L  A
        70        80        90       100       110       120
CCGACGTCTTCAACCCACAAGCCGGCCGAATCAACACCGTCGACAGCAACACTCTTCCAA
P  T  S  S  T  H  K  P  A  E  S  T  P  S  T  A  T  L  F  Q
       130       140       150       160                 180
TCCTCGATTTCCTCCAACTTAGCGCTCAGCACGTCGTCCTCTACAAGGTATAAACCCTAA
S  S  I  S  S  N  L  A  L  S  T  S  S  S  T  R
       190       200       210       220       230       240
TCCCCCAATTTGAACCATAAAATCGATTAATCATCAAATTGAACTTCACATTGGATGAAT

250       260       270       280       290       300
TAATTTTTATAGAATGCGATCCTCGGACCGAGATGGAACCTGAACGCACACAGCGCACTG
               N  A  I  L  G  P  R  W  N  L  N  A  H  S  A  L
       310       320       330       340       350       360
TACGTGACGAGAGGAGAAGGAAGAGTCCAGGTTGTCGGAGACGAAGGAAACGCAGTGTTC
Y  V  T  R  G  E  G  R  V  Q  V  V  G  D  E  G  N  A  V  F
       370       380       390       400       410       420
GACGATGTCGTCCAGAGAGGACAGATCCTGGTAGTCCCACAGGGATTCGCGGTGGTGTTG
D  D  V  V  Q  R  G  Q  I  L  V  V  P  Q  G  F  A  V  V  L
       430       440       450       460       470       480
AGGAAGAGAAGAGAAGGATTGGTGGTGGAGTTGAAGAACAGTGACAACGCCGTTACAAGT
R  K  R  R  E  G  L  V  V  E  L  K  N  S  D  N  A  V  T  S
       490       500       510       520       530       540
CCGATCGCCGGAAAGACGTCGGTGTTGAACGCGATTCCGGTGGACGTTTTGGCCACCGCG
P  I  A  G  K  T  S  V  L  N  A  I  P  V  D  V  L  A  T  A
       550       560       570       580       590       600
TATGATATCTCGAAGCCGGAAGCATTCAAGTTGAAGAACGGAAGGAGGCAGGAGATTGAG
Y  D  I  S  K  P  E  A  F  K  L  K  N  G  R  R  Q  E  I  E
       610       620       630       640
GTTTTCAGGCCATTCCAGTCCCGAGATGAGAAGGA
V  F  R  P  F  Q  S  R  D  E  K
```

**FIGURE 5.** Nucleotide and deduced amino acid sequences of the genomic DNA encoding the 22-kDa protein in *F. gracilipes*. The primer pairs used to amplify the genomic DNA are underlined. The intron sequences are indicated by bold letters. The arrows indicate the characteristic GU..AG dinucleotide combinations of the intron sequence.

## DISCUSSION

Only 2 of the 14 species of buckwheat showed the absence of the 22-kDa protein after SDS-PAGE of total seed proteins. While *F. lineare* and *F. urophyllum* showed a total absence of the 22-kDa band, it was difficult to predict in the case of *F. gracilipes*. In some samples, a very faint band was visible, while in some it was not. A bulk seed sample is a composite seed sample that represents a mixture of phenotypes. Hence, the number of seeds per sample may influence the results. In the present investigation, in the case of wild and remote species, only 10 seeds could be bulked. This could have had a direct effect on the protein profile. To confirm the results from SDS-PAGE and to develop a simple system for screening buckwheat plants for allergenicity, a PCR-based system was developed.

```
F.g.22kDa    1 GGATTGGAGCAAGCGTTCTGCAACCTTAAATTCAGGCAAAACGTGAAC-AACCTGCTCGC  59
F.e.22kDa    1 GGATTGGAGCAAGCGTTCTGCAACCTGAAATTCAGGCAAAATGTTAACAGGCCTTCTCGC  60
               ************************** ************** ** ***   *** *****
F.g.22kDa   60 GCCGACGTCTTCAACCCACAAG-C-CG-GCC-GAATCAACACCGTCGACAGCAACACTC  115
F.e.22kDa   61 GCCGACGTCTTCAACCCA--CGCGCCGGT-CGT-ATCAACACCGTAGACAGCAACAATC- 115
               ******************   *   **   *   *********** ********** **
F.g.22kDa  116 TC--CAATCCTCG-ATTTCCTCCAACTTAGCGCT-CAGCACGTCGTCCTCTACAAGGT-A 170
F.e.22kDa  116 TCCCG-ATCCTCGAAT-TCATCCAACTTAGCG-CCCAGCACGTCGTCCTCTACAAGGTAA 172
               **   ******* ** ** ************   *********************** *
F.g.22kDa  171 --TAAACC----C-TAATCC------CCCAATTTGAA--C--CA--TAAAA-T--CG--A 206
F.e.22kDa  173 TGTAAACCCCAGCTGAAGCCCTAAAAGCG-AT-TGAACTTCGTATTGAA-ATTCAAGACC 229
                 ******    *  ** **       *  ** ****      *   ** * *   *
F.g.22kDa  207 TTA--AT-C----AT---CAA-A-----T-TG--A-ACTTCACATT--GGATGAA---T- 240
F.e.22kDa  230 -TAAAATCGACAAATCTGCAAAGTGAAGCTTGATTAACTTCAGATTGACGA-TAATGCGT 287
                ** **      **   ***          **    ****** ***    **  **
F.g.22kDa  241 -TAATTTTTA--TAGAATGCGATCCTCGGACCGAGATGGAACC-TGAACGCACACAGCGC 296
F.e.22kDa  288 TGAATT--TATGCAGAATGCGATCCTCGGACCGAGATGGAA-CTTGAACGCGCACAGCGC 344
                 ****  **   **************************** * ******* ********
F.g.22kDa  297 ACTGTACGTGACG-AGAGGAGAAGGAAGAGTCC-AGGTTGTCG-GAGACGAAGGAAACGC 353
F.e.22kDa  345 ACTGTACGTGA-CAAGAGGAGAAGGAAGAGTCCAA-GTTG-TGGGAGATGAAGGA--A-G 398
               ***********   ******************* * ****  * **** ******
F.g.22kDa  354 -AGTG-T-TCGACG---ATGTCGTCCAGAGAGGACAGATCC-TGGTA-GTCCCACAGGGA 405
F.e.22kDa  399 AAGTGTGTTCGACGACAACG-T-G-CAGCGAGGACAGATCCTT-G-TGGTCCCACAGGGA 453
                ****   ******   * *     *** ************ * *   ************
F.g.22kDa  406 TTCG-CGGTGGTGTTG-AG-GAAGAGAAGAGAAGG-ATTG--GTG-GTGGAGTTGAAGAA 458
F.e.22kDa  454 TTCGCA-GTGGTGTTGAAGGC-A-GGAAGAGAAGGAC-TGGAGTGGGTGGAGTTGAAGAA 509
               ****   ********* **   *  **********    **   *** **************
F.g.22kDa  459 -C-AGTGACAACGCCGTT-A-CAAGTCCGATCGCCG-GAAAGACG-TCGGTGTTGAAC-- 510
F.e.22kDa  510 CGAC--GACAACGCCA-TAACC-AGTCCGATTGCCGGGA-AGA-CTTCGGTGTTG--AGG 561
                     *********  * * * ******** **** ** ***   *********
F.g.22kDa  511 GCGATTC--CGGTGGAC-GT-TTTGGCC-A-CCGCGTATGATATCTCG--A-AGCCGGAA 561
F.e.22kDa  562 GCGA-TCCCT-GTGG-AGGTTCTT-GCCAACT-C-CTACGATATCTCGACGAA--G-GAA 612
               **** **    ****    **   ** *** *      ** *********    *    ***
F.g.22kDa  562 GCAT-TC-AAGTTGAAGAACGGAAGGAGGCAGGAGATTGAGG-TTTTCAGGCCATTCCAG 618
F.e.22kDa613 G-CTTTCAG-ATTGAAGAATG--G-GAGGCAGGAGGTTGAGGTCT-TCCGACCATTCCAG 666
               *  * **    ******** *    ********** ******  * ** * *********
F.g.22kDa 619 TCCCGAGATGAGAAGGA----------------------                     635
F.e.22kDa 667 TCCCGAGATGAGAAGGAGAGGGAGCGTTTCTCCATAGTTT                    706
```

**FIGURE 6.** Multialignment pattern of nucleotides encoding the 22-kDa protein from *F. gracilipes* (F.g. 22 kDa) and *F. esculentum* (F.e. 22 kDa).

The strategy was to design primers that effectively amplify the region encoding the major allergenic protein. The results indicated that it is possible to achieve this. PCR is finding increasing application as a specific and sensitive diagnostic method for detection of food-borne pathogens[12,13,14]. One of the main limiting factors could be the primer pair, as they are very short sequences. However, the primer set BWA 17 and BWA 20 and the primer set BWA 18 and BWA 20 amplified the correct fragment of FAGAG1, the gene encoding the major buckwheat allergenic protein (DDBJ Accession no. AF 152003[15]) and resulted in one PCR product, making them applicable for screening.

Sequencing of PCR products from both *F. esculentum* and *F. gracilipes* gave an insight into the structure of the gene encoding the major allergenic protein. A 130-bp intron sequence was detected in the case of *F. esculentum* and an 80-bp intron sequence in the case of *F. gracilipes*. The plant introns contain characteristic /GU…AG/ dinucleotide combinations at their intron/exon boundaries. In comparison with mammalian transcripts, plant transcripts contain shorter introns (averaging < 150 bp) and longer exons[16]. The genomic DNA sequences of both *F. esculentum* and *F. gracilipes* showed the characteristic GU…AG dinucleotide combinations at their intron boundaries. The intron size was also less than 150 bp, adding credence to the data recorded in this study.

Although the same primer sets amplified the DNA sequence from both *F. esculentum* and *F. gracilipes*, considerable differences in their internal sequences were observed. As a result, the specific amino acids coding the 22-kDa protein were also different, ultimately leading to structural differences between the two proteins. Immunoblotting of *F. gracilipes* proteins using buckwheat allergic patients' sera revealed that the *F. gracilipes* proteins are not allergenic (Urisu, personal communication). Combining this result with the DNA sequence data of *F. gracilipes*, the absence of allergenicity could be correlated to the structural differences of the 22-kDa major allergenic protein from both *F. esculentum* and *F. gracilipes*. The differences in their respective protein structures may result in the alteration of immunodominant epitopes, making the *F. esculentum* protein allergenic and the *F. gracilipes* protein nonallergenic.

As regards *F. lineare*, the amplification of genomic DNA with the same set of primers failed to produce any band on agarose gel, indicating the usefulness of this method in screening different species or populations of buckwheat for allergenic proteins. Such allergen-deficient lines could be advanced through conventional breeding via interspecific hybridization and selection or through biotechnological approaches like protoplast fusion and regeneration. The PCR-based screening described in this article could also be successfully used in rapid screening of hypoallergenic transgenic plants.
